# Spectrum of paired‐like homeobox 2b immunoexpression in pediatric brain tumors with embryonal morphology

**DOI:** 10.1111/pin.13255

**Published:** 2022-06-28

**Authors:** Murad Alturkustani, Adam D. Walker, Ivy Tran, Matija Snuderl, Jennifer A. Cotter

**Affiliations:** ^1^ Department of Pathology King Abdulaziz University Jeddah Saudi Arabia; ^2^ Department of Pathology and Laboratory Medicine Children's Hospital Los Angeles Los Angeles California USA; ^3^ Department of Pathology University of Western Ontario London Ontario Canada; ^4^ Department of Pathology New York University Langone Medical Center New York New York USA; ^5^ Department of Pathology, Keck School of Medicine University of Southern California Los Angeles California USA

**Keywords:** brain tumors, CNS embryonal tumor, embryonal tumor with multilayered rosettes, immunohistochemistry, PHOX2B

## Abstract

Paired‐like homeobox 2b (PHOX2B) is an established immunomarker for peripheral neuroblastoma and autonomic nervous system cells. We aimed to evaluate the utility of PHOX2B immunostaining in central nervous system (CNS) tumors with embryonal morphology. Fifty‐one tumors were stained with PHOX2B and submitted for whole slide image analysis: 35 CNS tumors with embryonal morphology (31 CNS embryonal tumors and four gliomas); and 16 peripheral neuroblastomas were included for comparison. Diffuse nuclear immunopositivity was observed in all (16/16) neuroblastomas (primary and metastatic). Among CNS embryonal tumors, focal immunoreactivity for PHOX2B was observed in most (5/7) embryonal tumors with multilayered rosettes (ETMR) and a single high‐grade neuroepithelial tumor (HGNET) with *PLAGL2* amplification; the remaining 27 CNS tumors were essentially immunonegative (<0.05% positive). Among ETMR, PHOX2B expression was observed in a small overall proportion (0.04%–4.94%) of neoplastic cells but focally reached up to 39% in 1 mm ‘hot spot’ areas. In the *PLAGL2*‐amplified case, 0.09% of the total neoplastic population was immunoreactive, with 0.53% in the ‘hot spot’ area. Care should be taken in interpreting PHOX2B immunopositivity in a differential diagnosis that includes metastatic neuroblastoma and CNS tumors; focal or patchy expression should not be considered definitively diagnostic of metastatic peripheral neuroblastoma.

**Abbreviations**
ATRT: atypical teratoid rhabdoid tumors
CHLA: Children's Hospital Los Angeles
CIC: capicua transcriptional repressor
CMA: chromosomal microarray analysis
CNS: central nervous system
EFT: Ewing sarcoma family tumor
EGFR: epidermal growth factor receptor
ET: embryonal tumor
ETANTR: embryonal tumor with abundant neuropil and true rosettes
ETMR: embryonal tumors with multilayered rosettes
FOXR2: forkhead box R2
HGNET: high‐grade neuroepithelial tumor
NB FOXR2: neuroblastomas with *FOXR2* activation
NEC: not elsewhere classified
NOS: not otherwise specified
PHOX2B: paired‐like homeobox 2b
PNET: primitive neuroectodermal tumor

## INTRODUCTION

Neurocytic and ganglionic differentiation is a common feature among central nervous system (CNS) embryonal tumors and gliomas.^1^, ^2^, ^3^, ^4^ In some of these tumors, like CNS (ganglio)neuroblastomas and embryonal tumor with multilayered rosettes (ETMR), neurocytic and ganglionic cells may compose a significant proportion of the tumor. When these features are present in combination with a population of undifferentiated cells, some pediatric CNS tumors may closely resemble peripheral neuroblastoma, a pediatric malignancy which can metastasize to the brain.

The *PHOX2B* gene is a paired‐like homeobox gene which maps to 4p13 and encodes a transcription factor important for neural crest development and differentiation to sympathetic neurons.^5^ PHOX2B is considered highly specific for the peripheral autonomic nervous system, and diffuse strong nuclear immunoreactivity for PHOX2B has been established as a reliable marker for peripheral neuroblastoma and paraganglioma.^6^, ^7^ However, only limited studies have evaluated the pattern and extent of PHOX2B immunoreactivity in CNS tumors.^8^, ^9^ In this study, we investigated the utility of PHOX2B immunohistochemistry in a variety of CNS tumors with embryonal morphology.

## MATERIALS AND METHODS

This study was approved by the Institutional Review Board at Children's Hospital Los Angeles (CHLA), with a waiver of patient consent. The CHLA archives were searched for tumors originally diagnosed as either primitive neuroectodermal tumor (PNET) or CNS embryonal tumor at the time of initial review. The integrated diagnoses of these cases incorporated findings of subsequent molecular testing, in some cases changing the final assigned category of tumor. Orthogonal molecular testing results available for the cases included a mixture of next generation sequencing of DNA and RNA with the OncoKids® cancer panel, chromosomal microarray analysis (CMA), and clinically validated whole genome DNA methylation array with molecular classification,^10^ as shown in Table 1. All cases were reviewed by two neuropathologists (J.A.C. and M.A.) to confirm the integrated diagnoses and ensure adequacy of tissue for immunohistochemistry. Additionally, 34 cases of well‐defined tumors were added for comparison. These included 16 neuroblastomas (eight primary peripheral neuroblastomas and eight brain metastatic peripheral neuroblastomas), five atypical teratoid rhabdoid tumors (ATRT), six medulloblastomas, and seven pineoblastomas. We performed immunostaining with anti‐PHOX2B rabbit antibody (Abcam; clone no.: EPR14423, dilution of 1:100) for representative sections from each of the 51 tumors. Immunostained slides were digitized on an Aperio AT2 whole slide scanner.

**Table 1:** Clinicopathologic features of included cases and associated calculated paired‐like homeobox 2b (PHOX2B) percent positivity

No.	Age	Gender	Location	Integrated diagnosis	Pertinent histologic/molecular results	Positivity percentage	
						Overall	Hot spot
1	2Y	F	Brainstem	ETMR, C19MC‐altered	CMA: gain of chromosome 2 gain of 19q13.42 (C19MC)	0.6481	4.18
2	3Y	F	Parietal lobe	ETMR, NOS	LIN28 immunostain	0.0374	N/A
3	9W	F	Posterior fossa	ETMR, C19MC‐altered	CMA: gain of chromosome 2 gain of 19q13.42 (C19MC)	0.0473	N/A
4	18mo	F	Cerebellopontine angle	ETMR, C19MC‐altered	CMA: gain of chromosome 2 amplification of 19q13.42 (C19MC)	3.265	38.53
5	14mo	F	Brainstem	ETMR, C19MC‐altered	CMA: gain of chromosome 2 amplification of 19q13.42 (C19MC)	0.461	3.734
6	2Y	F	Frontal lobe	ETMR, NOS	LIN28 immunostain	0.7755	16.4
7	3Y	M	Frontal lobe	ETMR, C19MC‐altered	CMA: gain of chromosome 2 gain of 19q13.42 (C19MC)	4.939	39.01
8	4mo	M	Lateral ventricle	CNS embryonal tumor NEC (HGNET with PLAGL2 amplification)	CMA: PLAGL2 amp, MP‐CS: 0.999	0.085	0.5272
9	8Y	F	Temporal lobe	Diffuse pediatric‐type high‐grade glioma, MYCN amplified	CMA: EGFR, MDM4, and MYCN amplifications	0.0235	N/A
10	10Y	F	Frontal lobe	CNS NB, FOXR2 activated	CMA: 1q gain and 16q loss, MP‐CS: 0.991	0.015	N/A
11	17mo	F	Parietal lobe	CNS NB, FOXR2 activated	CMA: 1q gain and 16q loss, MP‐CS: 0.999	0.0066	N/A
12	3Y	F	Frontal lobe	CNS NB, FOXR2 activated	MP‐CS: 0.998	0.0227	N/A
13	7Y	M	Temporal lobe	CNS embryonal tumor NEC (methylation class CNS CIC‐rearranged sarcoma)	MP‐CS: 0.989	0.0373	N/A
14	4Y	M	Temporal lobe	CNS tumor with BCOR internal tandem duplication	BCOR ITD by fragment analysis	0.0314	N/A
15	16Y	F	Parieto‐occipital region	Astroblastoma, MN1‐altered	CMA: segmental copy number loss (deletion) at 22q11.23 to q12.1 and Xp22.33 to p22.13, consistent with MN1::BEND2 fusion, MP‐CS: 0.996	0.0098	N/A
16	16Y	M	Frontal lobe	Diffuse hemispheric glioma, H3 G34‐mutant	H3‐3A G34 mutant	0.0122	N/A
17	14Y	M	Fronto‐parietal region	Diffuse hemispheric glioma, H3 G34‐mutant	H3‐3A G34 mutant	0.0331	N/A
18	17mo	M	Posterior fossa	ATRT	INI1 negative	0.0027	N/A
19	18mo	M	Parietal lobe	ATRT	INI1 negative	0.0048	N/A
20	2Y	M	Pineal region	ATRT	INI1 negative	0.019	N/A
21	3Y	F	Frontal lobe	ATRT	INI1 negative	0.0298	N/A
22	2Y	F	Pineal region	ATRT	INI1 negative	0.004	N/A
23	4Y	F	Posterior fossa	MB, Group 3	MP‐CS: 0.999	0.003	N/A
24	7Y	F	Posterior fossa	MB, Group 4	MP‐CS: 0.971	0.009	N/A
25	17Y	M	Posterior fossa	MB, SHHA	MP‐CS: 0.987	0.0099	N/A
26	15mo	M	Posterior fossa	MB, SHHB	MP‐CS: 0.983	0.0292	N/A
27	6Y	M	Posterior fossa	MB, WNT	MP‐CS: 0.999	0.0304	N/A
28	10Y	F	Posterior fossa	MB, WNT	MP‐CS: 0.999	0.0136	N/A
29	2mo	M	Right abdomen	NB, poorly diff	OncoKids negative	45.9	90.58
30	20Y	F	Left paraspinal	NB, residual/recurrent	None	10.41	94.51
31	14mo	F	Right adrenal	NB, post‐treatment	None	1.819	38.48
32	5Y	M	Right adrenal	GNB, poorly diff	None	77.43	99.11
33	15mo	M	Abdomen	NB, poorly diff	None	31.9	12.51
34	4Y	M	Right adrenal	GNB, intermixed	None	2.455	96.51
35	11mo	M	Abdomen	NB, poorly diff	OncoKids negative	68.95	97.08
36	3mo	F	Paraspinal	NB, post‐treatment	None	9.429	55.77
37	2Y	F	Brain	Metastatic NB	None	66.06	99.22
38	16mo	M	Brain	Metastatic NB	ALK mutation	33.65	89.67
39	3Y	M	Brain	Metastatic NB	ALK mutation, MYCN amp	7.335	85.41
40	20mo	F	Dura	Metastatic NB	MYCN amp	79.22	88.57
41	2Y	F	Skull	Metastatic NB	None	46.51	90.67
42	3Y	F	Skull	Metastatic NB	None	1.947	7.56
43	3Y	M	Brain	Metastatic NB	ALK mutation, MYCN amp	29.38	97.2
44	2Y	M	Brain	Metastatic NB	None	63.6	97.15
45	3Y	F	Pineal region	Pineoblastoma	None	0.0073	N/A
46	10Y	F	Pineal region	Pineoblastoma	None	0.0353	N/A
47	11Y	F	Pineal region	Pineoblastoma	None	0.0376	N/A
48	16Y	M	Pineal region	Pineoblastoma	None	0.028	N/A
49	3Y	M	Pineal region	Pineoblastoma	None	0.035	N/A
50	4Y	F	Pineal region	Pineoblastoma	None	0.0104	N/A
51	8mo	M	Pineal region	Pineoblastoma	None	0.0148	N/A

*Note*: Cases with less than 0.05% overall positivity were not submitted for hot spot analysis (N/A).

Abbreviations: ALK, anaplastic lymphoma kinase; ATRT, atypical teratoid rhabdoid tumor; BCOR, BCL6 Corepressor; CIC, capicua transcriptional repressor; CMA, chromosomal microarray; CNS, central nervous system; C19MC, chromosome 19 microRNA cluster; EGFR, epidermal growth factor receptor; ETMR, embryonal tumors with multilayered rosettes; F, female; FOXR2, forkhead box R2; GNB, ganglio neuroblastoma; HGNET, high‐grade neuroepithelial tumor; INI1, integrase interactor 1; ITD, internal tandem duplication; M, male; MB, medulloblastoma; mo, months; MP‐CS, methylation profile, calibrated score for diagnosis; NB, neuroblastoma; NEC, not elsewhere classified; NGS, next generation sequencing; NOS, not otherwise specified; PLAGL2, pleomorphic ademona gene like‐2; SHHA, sonic hedgehog protein A; W, weeks; Y, years.

Quantification of percent positivity was performed using whole slide image analysis. Each digital slide was visually inspected. Areas of artifactual positivity (e.g., hemosiderin deposition, tissue folding, and other staining artifacts) were excluded from the region of analysis. Overall slide positivity was calculated, and for each slide that had an overall positivity above 0.05%, a 1 mm ‘hot spot’ was computed using the image analysis tool Qupath in conjunction with a custom‐made Python script.^11^ Cells were classified using Qupath's positive cell detection algorithm, and, using cell meta‐data from Qupath, cell‐cell distance was calculated using Python's SciPy package. The cell data was downsampled by a factor of four to allow for more efficient processing. The positive cell with the greatest number of positive neighbors within a 500 μm radius was determined to be the center of the ‘hot spot.’ Image processing was completed on a Lenovo Thinkstation P340 with an Intel® Xeon® W‐1290P CPU at 3.70 GHz, 3696 Mhz, 10 core(s), 20 logical processor(s), and 32 GB of RAM. Initial cell identification was processed in Qupath (version 0.3.2). Hot spot analysis was developed in Python (version 3.10). In this analysis, we used Pandas (version 1.4), NumPy (version 1.22), SciPy (version 1.8), Argparse (version 1.1), and os. The Groovy code for drawing the hot spot was written within Qupath (version 0.3.2). All codes can be found at https://github.com/a-dev-walker/CentroidParser. Results of automated analysis were manually reviewed to ensure true nuclear positivity and ensure against artifactual false positive signals.

The final study cohort included 51 total tumor cases (Table 1). Cases included 31 CNS embryonal tumors (six CNS medulloblastomas, seven CNS pineoblastomas, five CNS ATRT, seven embryonal tumors with multilayered rosettes (ETMR), three CNS neuroblastomas with *FOXR2* activation (CNS NB FOXR2), one CNS tumor with BCL6 Corepressor (BCOR) internal tandem duplication, and two CNS embryonal tumors not elsewhere classified (NEC) (one with *PLAGL2* amplification and one with a methylation class match to *CIC*‐rearranged sarcoma, but lacking corresponding copy number alterations)); one astroblastoma, *MN1*‐altered; two cerebral gliomas with H3‐3A G34R mutation; one pediatric‐type high‐grade glioma with MYCN amplification; and 16 peripheral neuroblastomas (eight primary neuroblastomas and eight brain, dura, or skull‐based metastatic neuroblastomas).

The morphology of 5/7 ETMR cases corresponded to the embryonal tumor with abundant neuropil and true rosettes (ETANTR) pattern. All five ETANTR pattern ETMR cases showed at least focal immunostaining with anti‐LIN28 antibody, gain of chromosome 2 and the characteristic gain or amplification of 19q13.42 that includes the micro RNA (miRNA) cluster referred to as C19MC. The remaining two cases of ETMR (Cases 2 and 6) showed histologic features of medulloepithelioma and ependymoblastoma respectively and had focal immunoreactivity with anti‐LIN28 antibody.

The included four glioma cases were initially diagnosed as CNS PNET and were revised after molecular testing confirmed their glial diagnoses. Specifically, CMA of Case 9 showed gain of chromosome 7 and amplifications of strong clinical significance in *EGFR*, *MDM4*, and *MYCN*, as described in previous work.^12^ The *H3‐3A* G34R mutation was confirmed by immunohistochemistry and sequencing in Cases 16 and 17. Diagnosis of Case 15 as astroblastoma, *MN1*‐altered, was based on copy number alterations consistent with *MN1::BEND2* fusion and an aligned DNA methylation profile. The diagnosis of CNS tumor with BCOR internal tandem duplication (ITD) was based on the morphological features of high‐grade astrocytic neoplasm and the detection of BCOR ITD by fragment analysis.

The diagnoses of three cases of CNS NB FOXR2 were based on characteristic copy number alterations (gain of 1q and loss of 16q) as well as aligned DNA methylation profiles. One of the cases diagnosed as CNS embryonal tumor NEC had a *PLAGL2* amplification (Case 8) as demonstrated by chromosomal microarray and an aligned methylation profile with the recently added category of ‘HGNET with PLAG‐family amplification’ from v12.3 of the DKFZ brain classifier accessible at molecularneuropathology.org.^10^ The other case diagnosed as CNS embryonal tumor NEC showed a high score class match to ‘CNS Ewing sarcoma family tumor with CIC alteration (CNS EFT‐CIC)’ on version 12.3 of the DKFZ brain classifier; however, corresponding copy number changes were not identified, and RNA sequencing was not available.

For comparative analysis, classic cases of ATRT, medulloblastoma, and pineoblastoma were included in the cohort. Relevant clinical, histologic, and molecular features of these tumors are described in Table 1. In addition, classic peripheral neuroblastoma/ganglioneuroblastoma cases (eight primary peripheral neuroblastomas and eight metastatic peripheral neuroblastomas) were included based on the established utility of PHOX2B in this tumor category.^6^, ^7^

## RESULTS

**Figure 1:**
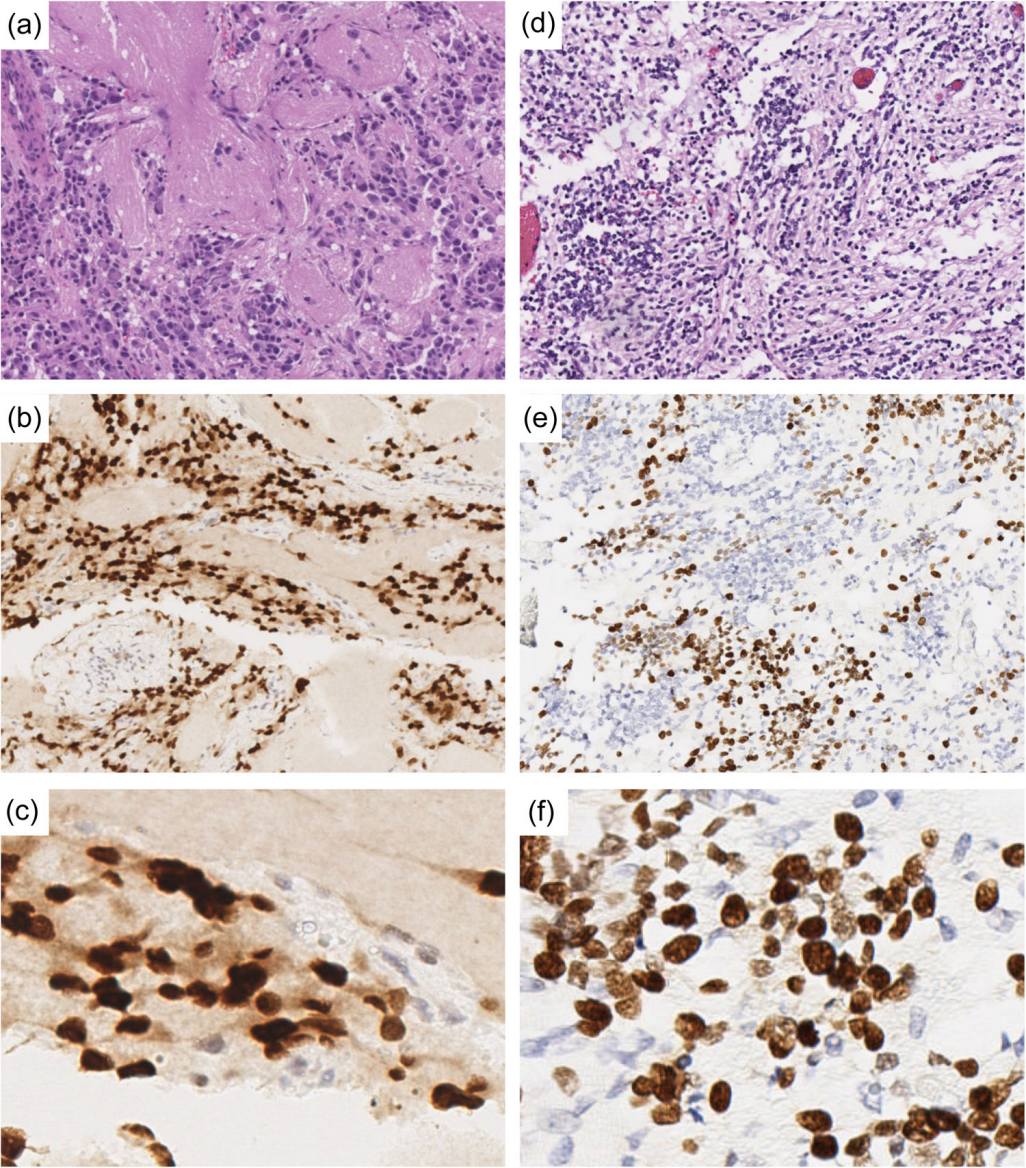
Pattern of paired‐like homeobox 2b (PHOX2B) expression usually differentiates metastases of peripheral neuroblastoma from central nervous system tumors with embryonal morphology, but focal elevated percent positivity is present in some embryonal tumors with multilayered rosettes (ETMR). (a–c) Case 38, peripheral neuroblastoma metastatic to the brain (a: hematoxylin and eosin (H&E) of area with neurocytic and ganglion cell differentiation, 100× magnification; b: PHOX2B showing a diffuse pattern of immunoreactivity, 100× magnification; c: PHOX2B, 400× magnification). (d–f) Case 7, embryonal tumor with multilayered rosettes (d: H&E of neuropil‐rich area with neuronal differentiation, 100× magnification; e: PHOX2B showing scattered small aggregates of positive cells, 100× magnification; f: PHOX2B, 400× magnification).

Clinical features, integrated diagnoses, and percent positivity are summarized in Table 1. All cases showed at least a single immunoreactive cell (positivity > 0%). The adjacent reactive or normal brain tissue, when present, did not show evidence of PHOX2B immunoreactivity, and no immunoreactivity was present in vascular endothelial cells. All 16 peripheral neuroblastomas, including eight cases with intracranial metastasis, showed widespread nuclear staining for PHOX2B (Figure 1), with overall percent positivity ranging from 1.8% to 79.2%. All neuroblastomas had a hotspot positivity exceeding 7.5%.

**Figure 2:**
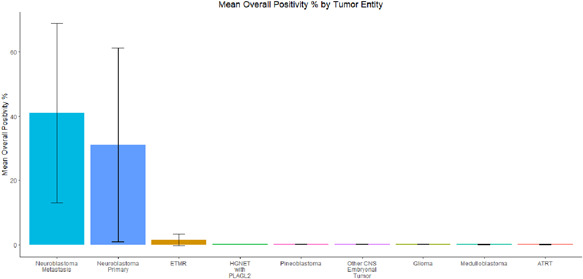
Mean overall paired‐like homeobox 2b (PHOX2B) percent positivity by tumor entity. Primary and metastatic neuroblastomas showed more widespread PHOX2B immunoreactivity than all central nervous system (CNS) tumors. Among CNS tumors, embryonal tumors with multilayered rosettes (ETMR) showed the highest mean overall PHOX2B immunoreactivity. ‘Other CNS embryonal tumor’ includes Cases 8, 10–12, and 14. ‘Glioma’ includes Cases 9 and 15–17.

**Figure 3:**
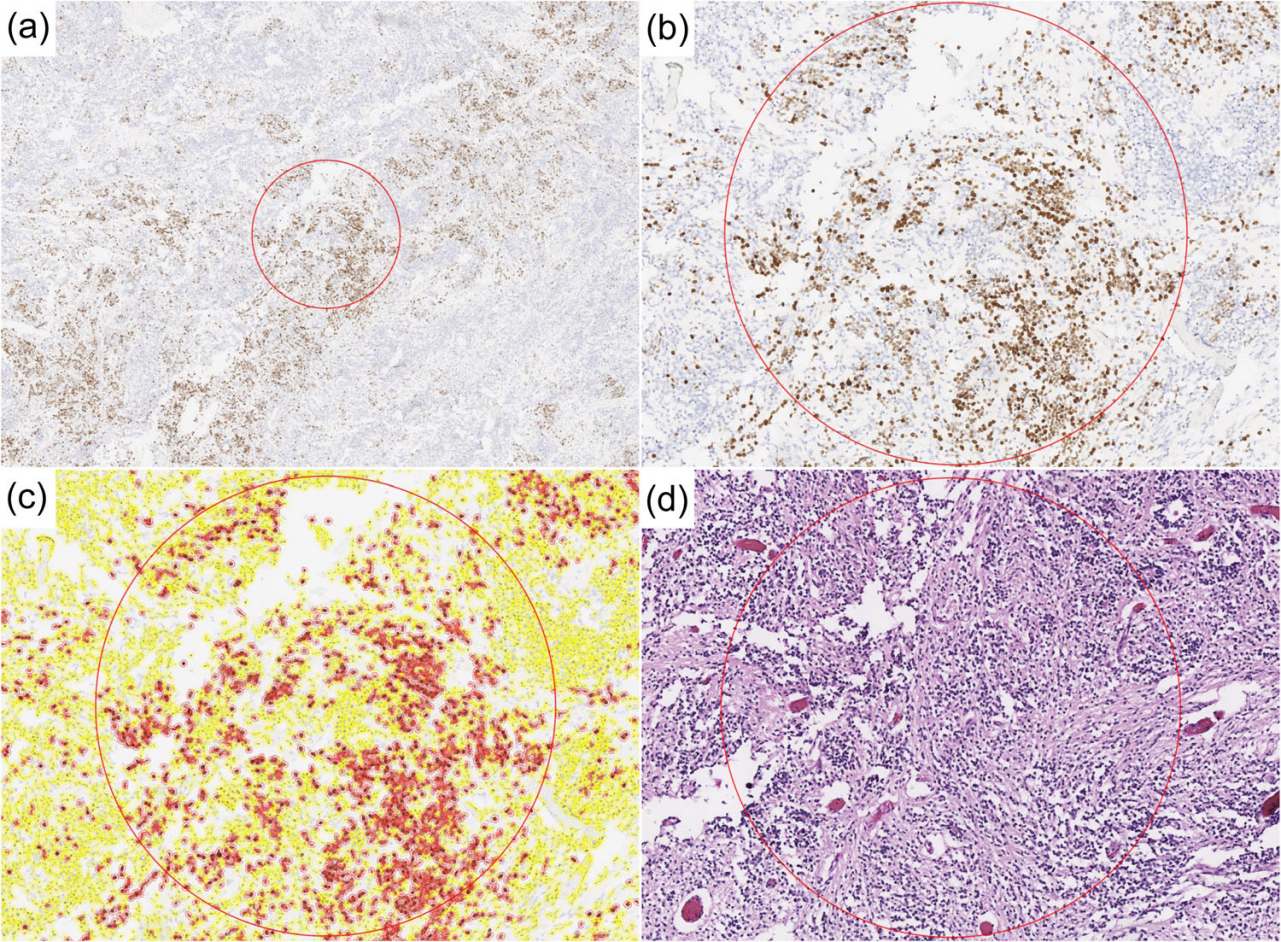
Some embryonal tumors with multilayered rosettes (ETMR) contain ‘hot spots’ exceeding 20%–30% paired‐like homeobox 2b (PHOX2B) positivity. (a–d) Determination of ‘hot spot’ in the ETMR with highest overall percent expression (Case 7). (a) The PHOX2B immunostain whole slide image was analyzed by the program to determine the overall positivity and the highest expression ‘hot spot’ 1 mm area, circled. (b) ‘Hot spot,’ enlarged. The center of the ‘hot spot’ was defined as the PHOX2B‐positive cell with the greatest number of positive neighbors within a 500 μm radius. (c) Color overlay indicated what the program recognized as positive (red) and negative (yellow) cells. (d) Corresponding hematoxylin and eosin (H&E) of this area, showing that areas of high PHOX2B percent positivity corresponded to less cellular, neuropil‐rich regions.

Overall, none of the CNS tumors in our study demonstrated diffuse strong nuclear expression of PHOX2B comparable to that expected in the setting of peripheral neuroblastoma (Figure 2). Nuclear immunoreactivity over 0.05% for PHOX2B was observed in most (5/7) ETMR cases and in the HGNET with *PLAGL2* amplification. The remainder of CNS tumor cases were immunonegative (<0.05%). Among ETMR cases, PHOX2B expression was observed in varying proportions of neoplastic cells ranging from 0.03% to 1.98% but reaching focally up to 37.19% in ‘hot spot’ areas (Figure 3). The irregular distribution of the PHOX2B immunopositivity among neoplastic cells accounts for the large discrepancy in the calculated overall immunopositivity versus ‘hot spot’ positivity among ETMR cases. Among ETMR cases, PHOX2B‐immunopositive neoplastic cells were more prominent in areas of apparent neuronal differentiation with neuropil background, and were less frequent in highly cellular, more undifferentiated appearing areas (Supporting Information: Figure S1). Multilayered rosettes occasionally contained scattered immunopositive cells.

In the CNS ET NEC *PLAGL2*‐amplified case, 0.09% of cells were immunoreactive overall, with 0.53% positive in the ‘hot spot’ area.

The included medulloblastoma, pineoblastoma, and ATRT cases all showed less than 0.05% overall positivity.

## DISCUSSION

Embryonal tumors of the CNS are a heterogenous group. They include well‐defined entities like medulloblastoma, atypical teratoid rhabdoid tumor, ETMR, and more recently defined entities emerging from the category previously termed PNET.^13^ Most of these entities are histologically characterized by a variable extent of neuronal differentiation ranging from neuronal marker‐positive undifferentiated neoplastic cells to histologically well‐differentiated neurons or ganglion cells. In addition, some subtypes of pediatric and young adult high‐grade gliomas, including those with *MN1* alterations, *MYCN* amplifications, and *H3‐3A* G34 mutations, may be histologically indistinguishable from CNS embryonal tumors.^2^ The common undifferentiated appearance and variable neuronal differentiation present in many pediatric CNS tumors can occasionally create histologic overlap with peripheral neuroblastoma.

In normal development, the *PHOX2B* gene is a key transcription factor important for neural crest development and differentiation to sympathetic neurons.^5^ *PHOX2B* is expressed in the early human embryo in the terminal rhombomeres 4‐8 in the hindbrain of the CNS and in many neural crest derivatives like sympathetic chain ganglia, the 7th, 9th and 10th ganglionic complexes,^14^ and neuro‐enteric ganglia in both neuronal and glial precursors.^15^ *PHOX2B* polyalanin expansion and frameshift mutations have been linked to CNS disease (i.e., congenital central hypoventilation syndrome) with neural crest‐derived tumors (neuroblastoma) and neural crest migration defects (Hirschsprung disease).^14^

Diffuse expression of PHOX2B is a characteristic feature of peripheral neuroblastoma, and its expression is reduced with more cellular differentiation, a feature that was observed in cases 31 (post‐treatment) and 34 (ganglioneuroblastoma).^7^ In human neuroblastoma cell lines, retinoic acid administration induces cellular differentiation, which is accompanied by a decrease in *PHOX2B* expression, and in mouse models, a downregulation of *PHOX2B* inhibits neoplastic proliferation. High PHOX2B expression in neoplastic cells is associated with poor prognosis.^16^

To date there has been limited study of PHOX2B staining in CNS tumors. Alexandrescu et al. reported PHOX2B immunoreactivity of less than 1% in 4/4 ETMR and 2/4 ATRT, as well as immunoreactivity in 3/6 pineoblastomas (two with 40%–50% immunopositivity).^8^ Other tumor types in that study included retinoblastomas, medulloblastomas, and CNS embryonal tumor NEC, which all demonstrated rare or no immunoreactivity (*n* = 17). Ma et al. found no PHOX2B staining in all 210 CNS tumors they examined which included 10 pineoblastomas and three ETMR cases.^9^ These results differ from the partial immunoreactivity observed by Alexandrescu et al. in pineoblastomas^8^ and from our observation of partial immunoreactivity in ETMR. Our finding of lack of significant PHOX2B expression in pineoblastomas is concordant with Ma et al., and disconcordant with Alexandrescu et al.

We used the same antibody clone as these two other studies, but our standard clinical dilution of 1:100 is significantly more concentrated than the Ma et al. study (1:1000), and slightly more concentrated than Alexandrescu et al (1:125) which could influence the extent and strength of observed staining. In agreement with the previous findings of both studies, none of the examined CNS tumor cases in our study showed the pattern of widespread nuclear PHOX2B immunoreactivity that would be expected in a peripheral neuroblastoma. However, as shown in our data, peripheral neuroblastomas can show a wide range of immunostaining ranging from negative to diffuse^7^; therefore, patchy or focal nuclear staining for PHOX2B should be considered nonspecific based on our findings.

Our study showed in 5/7 ETMR cases with at least rare immunoreactivity for PHOX2B. For the most part, the scattered single immunopositive cells observed in more differentiated areas could easily be overlooked by visual inspection. However, the presence of PHOX2B‐positive aggregates, creating ‘hot spots’ of higher percent positivity, up to 39.01%, has not been previously recognized as a feature of ETMR. The irregular distribution of this PHOX2B positivity in conjunction with sampling bias could potentially lead to misdiagnosis, and warrants caution.

The biological basis for PHOX2B immunoexpression in primary brain tumors, and the reason for its association with specific tumor types (i.e., ETMR and pineoblastoma) is unclear. One possible explanation is a sub‐clonal differentiation toward PHOX2B‐positive neurons, normally present in the embryonic terminal rhombomeres and the respiratory centers of the brain stem.^14^, ^17^ This hypothesis is supported by the fact that some malignant cells in ETMR show histologic features of neuronal differentiation, and that PHOX2B expression was mostly observed in neuropil‐rich areas in our study.^18^ Of note, extent of PHOX2B expression did not correlate to anatomic site; both supratentorial and infratentorial ETMR cases in our study showed focal PHOX2B expression. Alternatively, PHOX2B expression may indicate peripheral autonomic nervous system differentiation in a subset of the neoplastic cells. Differentiation is a common phenomenon in cancer cells^19^ which can include differentiation patterns typical of tissue outside the affected organ.

In our study, PHOX2B positivity was also noted in a single unusual case of CNS embryonal tumor NEC (a HGNET with *PLAGL2* amplification). *PLAGL2* was recently described as a regulator of *MYCN* expression in peripheral neuroblastoma, with knockdown of *PLAGL2* inducing neurite outgrowth and differentiation in neuroblastoma cells.^20^ High *PLAGL2* messenger RNA expression in neuroblastoma patient tumors also correlated to poor overall survival. *PLAGL2* is hypothesized to maintain neuroblastoma tumor cells in an undifferentiated state. Determination of whether there is any connection between *PLAGL2* amplification in this CNS embryonal tumor and expression of PHOX2B will require closer study.

PHOX2B expression focally exceeding 20%–30% of cells can be observed in ETMR, and focal PHOX2B expression in a subset of tumor cells was observed in a single case of CNS embryonal tumor NEC (HGNET with *PLAGL2* amplification). While focal positivity for PHOX2B has been previously reported in pineoblastoma, this finding was not observed in our cohort. The pattern of widespread PHOX2B immunoreactivity typical of peripheral neuroblastoma was not observed in any of the pediatric CNS tumors with embryonal features in our study. The underlying mechanism or genetic correlate for focal PHOX2B expression observed in the five positive ETMR cases and in the *PLAGL2*‐amplified case is not entirely clear but may correspond to neuronal differentiation among tumor cells. Care should be taken in the interpretation of focal PHOX2B positivity in any differential diagnosis including CNS embryonal tumors, especially in a small volume sample. Diffuse nuclear staining with PHOX2B in a small round blue cell tumor from the CNS is consistent with metastatic peripheral neuroblastoma. Focal or patchy nuclear staining with PHOX2B is not specific and can be observed in primary CNS tumors, particularly ETMR.

## AUTHOR CONTRIBUTIONS

Murad Alturkustani and Jennifer A. Cotter were responsible for conception and design of the study, acquisition and analysis of data and drafting the manuscript. Adam D. Walker scanned the slides, performed whole slide image analysis for the immunostained slides, wrote the code to perform ‘hot spot’ analysis, and drafted the manuscript. Matija Snuderl and Ivy Tran performed DNA methylation profiling of selected cases. All authors participated in the preparation of the manuscript.

## CONFLICT OF INTEREST

The authors declare no conflict of interest.

## Supporting information

Supplemental Figure 1: Paired‐like homeobox 2b (PHOX2B) immunopositive neoplastic cells are irregularly distributed in some central nervous system (CNS) tumors. A: The patchy distribution of PHOX2B immunopositive neoplastic cells highlights the importance of sampling. The left lower tissue fragment shows only scattered immunopositive cells, while the right upper fragment shows foci with frequent immunopositive cells. B‐C: Higher magnification of the latter area shows immunopositive cells are predominantly in the areas with neuropil background compared to the dense undifferentiated areas (A‐C: Case 4; original magnification, A: 20×, B: 100×, C: 400×). D: Rare PHOX2B immunopositive cells in area containing multilayered rosettes. (Case 1; original magnification: 400× E,F: PHOX2B immunopositive cells exceeding 0.05% of total cells were present in 22 of 51 cases). (Case 3; original magnification, E: 200×, F: 400×).
