# Supplementary figures and images for: Spectrum of paired‐like homeobox 2b immunoexpression in pediatric brain tumors with embryonal morphology
Source: Pathol Int. 2022 Jun 28;72(8):402–10. doi: 10.1111/pin.13255 (PMC9544136; doi:10.1111/pin.13255)

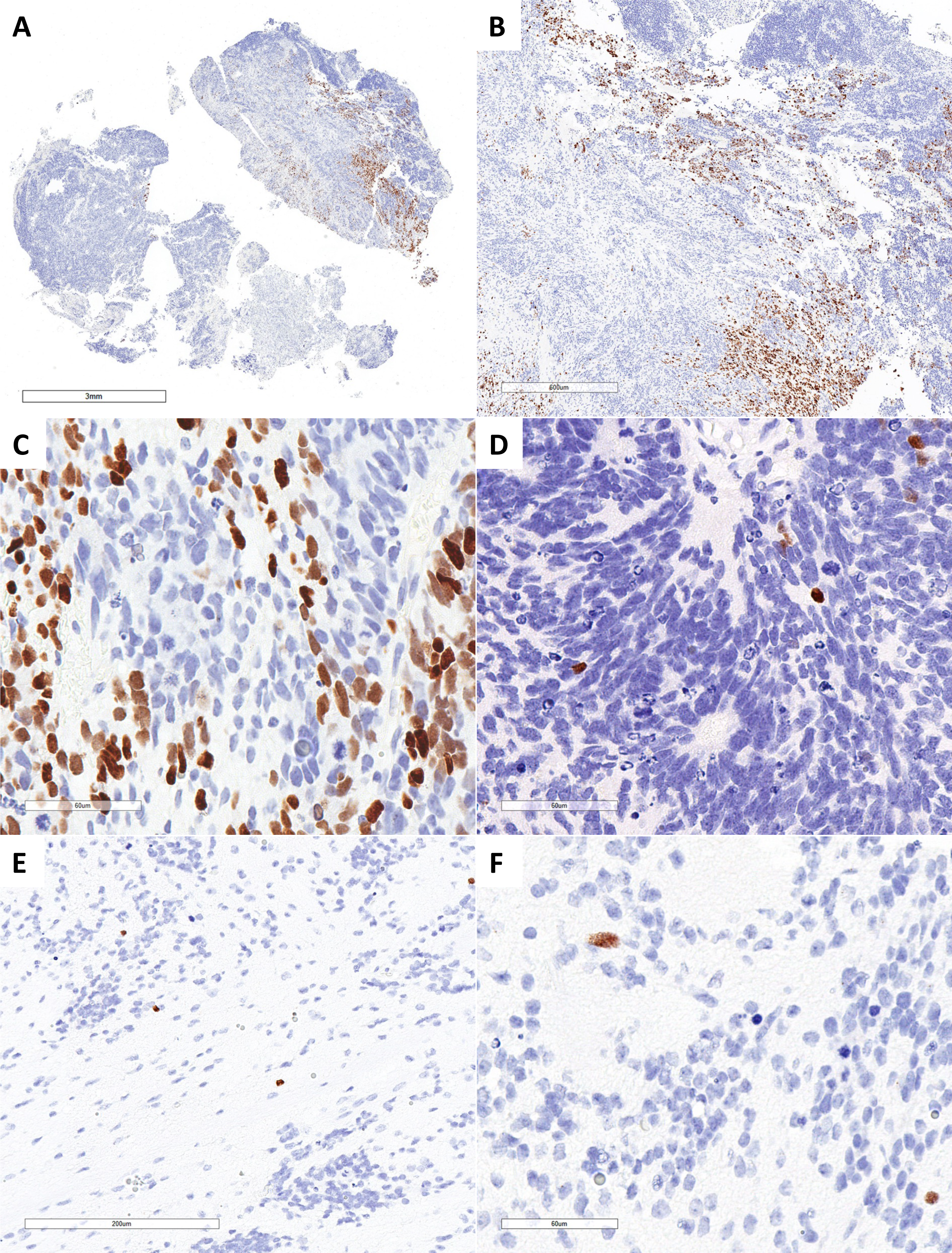

Supplement: Supplementary file 1 — Supplemental Figure 1: Paired‐like homeobox 2b (PHOX2B) immunopositive neoplastic cells are irregularly distributed in some central nervous system (CNS) tumors. A: The patchy distribution of PHOX2B immunopositive neoplastic cells highlights the importance of sampling. The left lower tissue fragment shows only scattered immunopositive cells, while the right upper fragment shows foci with frequent immunopositive cells. B‐C: Higher magnification of the latter area shows immunopositive cells are predominantly in the areas with neuropil background compared to the dense undifferentiated areas (A‐C: Case 4; original magnification, A: 20×, B: 100×, C: 400×). D: Rare PHOX2B immunopositive cells in area containing multilayered rosettes. (Case 1; original magnification: 400× E,F: PHOX2B immunopositive cells exceeding 0.05% of total cells were present in 22 of 51 cases). (Case 3; original magnification, E: 200×, F: 400×). [file PIN-72-402-s001.tif]
